# Supplementary material for: Correlation of preoperative frailty with postoperative delirium and one-year mortality in Chinese geriatric patients undergoing noncardiac surgery: Study protocol for a prospective observational cohort study
Source: PLoS One. 2024 Mar 6;19(3):e0295500. doi: 10.1371/journal.pone.0295500 (PMC10917300; doi:10.1371/journal.pone.0295500)
Supplement: S1 File — (DOC) [file pone.0295500.s001.doc]

**研究计划书**

**中国老年非心脏手术患者术前虚弱与术后谵妄和1年死亡率的相关性：一项单中心、前瞻性、观察性、队列研究**

**Correlation of preoperative frailty with postoperative delirium and one-year mortality in Chinese geriatric undergoing noncardiac surgery patients: study protocol for a prospective observational cohort study**

**负责单位： 山东第一医科大学第一附属医院**

**研究负责人：山东第一医科大学第一附属医院麻醉与围术期医学科**

**副主任医师 孙永涛**

**版本号：第四版 2021-11-8**

**中国老年非心脏手术患者术前虚弱与术后谵妄和1年死亡率的相关性：一项单中心、前瞻性、观察性、队列研究**

**Correlation of preoperative frailty with postoperative delirium and one-year mortality in Chinese geriatric undergoing noncardiac surgery patients: study protocol for a prospective observational cohort study**

1. **背景**

我国作为世界上老年人口最多的国家，也是人口老龄化发展速度最快的国家之一。预计到 2025 年，全国 65 岁以上人口总数将近 3 亿; 到 2050 年，我国将有 4 亿老年人。老龄化问题不断加重，更重要的是，过去 20 年接受外科手术的老年人

数量增长速度超过了人口老龄化的速度^［1］^。且有研究表明，与非手术老年人群( 虚弱比例占 4%～10%) 比较，虚弱在手术人群中更为普遍( 虚弱比例占 42%～50%) ，因此术前对老年患者综合健康状况的评估以及早逆转或减轻虚弱状态迫在眉睫^［2］^。因为每年有超过 50 万 65 岁以上的患者接受低风险和高风险手术^[3]^。

衰弱( frailty)是老年人因生理储备下降而出现抗应激能力减退的非特异性状态，涉及多系统的生理学变化，包括神经肌肉系统、代谢及免疫系统改变。是一种复杂的、多层面的、周期性的生理储备减少状态，导致恢复力和适应能力下降，对压力源的脆弱性增加。这种状态增加了死亡、失能、谵妄及跌倒等负性事件的风险^[4]^。 衰弱与围术期不良结局相关，被认为是导致某些心血管疾病（例如高血压、心力衰竭和缺血性心脏病）发展的危险因素之一。 衰弱普遍存在于老年手术患者^[5,6,7]^; 在70岁以上的老年人中，有38％～54％的人存在衰弱，综合衰弱程度为35％～41％。

术后谵妄（postoperative delirium, POD）是一种常见的并发症，通常发生于术后24 h至72 h，困扰着 20% 至 80% 的老年手术患者^[8]^。目前已知术后谵妄POD有多种可干预和不可干预的风险因素，如年龄、手术类型、入住ICU、疼痛和某些药物。年龄是不可干预的主要风险因素之一。因此，指南建议老年手术患者接受术前筛查，以筛查与术后谵妄和手术结果不佳相关的老年病^[9]^。其中最主要的是衰弱，衰弱还是是多种不良健康结局的有力预测因素，包括老年人的跌倒、失能和痴呆等^［10,11］^。同时，虚弱亦是手术预后的独立预测因子^［12］^，并被证明是导致老年人死亡的最常见疾病^[13]^，这表明在临床实践中识别老年患者的虚弱至关重要。因此，早期识别衰弱患者，对于术前决策以及预后评估均具有重要的临床意义。迄今为止，尚未评估非心脏手术老年患者术后谵妄的发生情况，本研究拟探讨术前衰弱评估与非心脏手术后术后谵妄发生率的关系。

**参考文献：**

1. Kulason Kay,Nouchi Rui,Hoshikawa Yasushi et al. Indication of Cognitive Change and Associated Risk Factor after Thoracic Surgery in the Elderly: A Pilot Study.[J] .Front Aging Neurosci, 2017, 9: 396.
2. Fried L P,Tangen C M,Walston J et al. Frailty in older adults: evidence for a phenotype.[J] .J Gerontol A Biol Sci Med Sci, 2001, 56: M146-56.
3. Schwarze ML, Barnato AE, Rathouz PJ, Zhao Q, Neuman HB, Winslow ER, Kennedy GD, Hu YY, Dodgion CM, Kwok AC, Greenberg CC. Development of a list of high-risk operations for patients 65 years and older. JAMA Surg. 2015 Apr;150(4):325-31. doi: 10.1001/jamasurg.2014.1819. PMID: 25692282; PMCID: PMC4414395.
4. Clegg A，Young J，Iliffe S，et al. Frailty in elderly people［J］. Lancet，2013，381（9868）： 752-762. DOI： 10.1016/S0140-6736（12）62167-9.
5. Woo J, Yu R, Wong M, Yeung F, Wong M, Lum C: Frailty screening in the community using the FRAIL scale. J Am Med Dir Assoc. 2015; 16:412–9
6. Gleason LJ, Benton EA, Alvarez-Nebreda ML, Weaver MJ, Harris MB, Javedan H: FRAIL questionnaire screening tool and short-term outcomes in geriatric fracture patients. J Am Med Dir Assoc. 2017; 18:1082–6
7. Aprahamian I, Cezar NOC, Izbicki R, Lin SM, Paulo DLV, Fattori A, Biella MM, Jacob Filho W, Yassuda MS: Screening for frailty with the FRAIL scale: A comparison with the phenotype criteria. J Am Med Dir Assoc. 2017; 18:592–6.
8. Inouye SK. Delirium in older persons. N Engl J Med. 2006 Mar 16;354(11):1157-65. doi: 10.1056/NEJMra052321.
9. Chow WB, Rosenthal RA, Merkow RP, Ko CY, Esnaola NF; American College of Surgeons National Surgical Quality Improvement Program; American Geriatrics Society. Optimal preoperative assessment of the geriatric surgical patient: a best practices guideline from the American College of Surgeons National Surgical Quality Improvement Program and the American Geriatrics Society. J Am Coll Surg. 2012 Oct;215(4):453-66. doi: 10.1016/j.jamcollsurg.2012.06.017. Epub 2012 Aug 21. PMID: 22917646.
10. Aldecoa C, Bettelli G, Bilotta F, Sanders RD, Audisio R, Borozdina A, Cherubini A, Jones C, Kehlet H, MacLullich A, Radtke F, Riese F, Slooter AJ, Veyckemans F, Kramer S, Neuner B, Weiss B, Spies CD. European Society of Anaesthesiology evidence-based and consensus-based guideline on postoperative delirium. Eur J Anaesthesiol. 2017 Apr;34(4):192-214. doi: 10.1097/EJA.0000000000000594. Erratum in: Eur J Anaesthesiol. 2018 Sep;35(9):718-719. PMID: 28187050.
11. Dent E，Martin FC，Bergman H，et al． Management of frailty: opportunities，challenges，and future directions［J］． Lancet，2019，394 ( 10206) : 1376-1386．
12. Amini S, Crowley S, Hizel L, Arias F, Libon DJ, Tighe P, Giordano C, Garvan CW, Enneking FK, Price CC. Feasibility and Rationale for Incorporating Frailty and Cognitive Screening Protocols in a Preoperative Anesthesia Clinic. Anesth Analg. 2019 Sep;129(3):830-838. doi: 10.1213/ANE.0000000000004190. PMID: 31425227; PMCID: PMC6927245.
13. Gill TM, Gahbauer EA, Han L, Allore HG. Trajectories of disability in the last year of life. N Engl J Med. 2010 Apr 1;362(13):1173-80. doi: 10.1056/NEJMoa0909087. PMID: 20357280; PMCID: PMC2877372.
14. **研究目的**
15. 研究衰弱与老年非心脏手术患者术后谵妄是否存在相关性
16. 改良衰弱指数能否预测非心脏手术术后的谵妄
17. 衰弱指数中哪些是与术后谵妄相关的独立危险因素

**三、入组标准**

1. 年龄≥70岁；
2. ASA：Ⅰ～Ⅳ级；
3. 签署知情同意书；
4. 计划择期行非心脏手术。

**四、排除标准**

1. 拒绝参加；
2. 预期住院时间<3天；
3. 同一患者只能纳入1次，无论二次手术原因是否与第一病因有关；
4. 急诊手术患者；
5. 文盲、语言障碍、严重的听力或视力损害而无法进行交流；
6. 中枢神经系统疾病，包括各种类型痴呆、抑郁症
7. 严重肾功能不全(需要透析治疗) ；
8. 严重肝功能异常(Child-pugh 评分≥10 分)；
9. 3个月内参加过其他相关临床研究的患者
10. MMSE 检查证实己存在认知功能异常者：文盲≤17分，小学程度≤20分，中学程度（包括中专）≤22分，大学程度（包括大专）≤23分；

**五、脱落标准**

病人或者委托人拒绝知情同意或者要求撤出该研究。

**六、研究类型**

**该研究是一项单中心、前瞻性、****观察性、队列研究**

1. 对于每个招募的患者，各研究员术前1天完成术前评估：衰弱筛查、认知功能评估、术前数据收集；
2. 按术前访视分工，各研究人员将负责术中指标收集，标本采集（术前、术毕）等；
3. 另外3名研究人员将负责术后1-3d随访、数据收集；
4. 统计分析将由另外指定的统计学家独立进行。

**七、样本量估算**

依据预实验≥70岁非心脏手术患者，衰弱发生率23%；衰弱组POD发生率为35.7%，非衰弱组POD发生率21.7%。采用PASS 15软件两组独立样本率比较的样本量计算方法：设实验组与对照组样本量之比0.303，双侧检验，检验水准（α）和检验效能（1-β）分别设定为0.05和0.80，并考虑10%脱落率，计算衰弱组样本量为114例，对照组样本量375例，共489名患者。

八、**全身麻醉**

麻醉诱导均使用力月西0.05-0.1 mg/kg，丙泊酚1.5-2 mg/kg或依托咪酯0.15-0.3mg/kg，舒芬太尼0.3-0.5μg/kg，罗库溴铵0.6 mg/kg或顺式阿曲库铵0.15 mg/kg。麻醉维持使用丙泊酚1.5-4.0μg/mL维持，阿片类药物采用瑞芬太尼0.1-2μg/(kg·min)维持，肌松药采用罗库溴铵5-10μg/kg/min维持。维持呼吸末CO2分压35～45 mmHg、脑电双频指数（Bispectral index, BIS）麻醉深度40～60。术中给予昂丹司琼8mg。术后给予患者静脉自控镇痛（PCIA，舒芬太尼0.08-0.1μg/kg/h，总量控制在150-250 μg以内）。

**九、术前数据**

各研究员认真完成衰弱评估与日常生活能力评估（评分标准见附件1）。

1. 日常生活能力评估：术前1天，利用Barthel指数评定表对患者进行日常生活能力评估；0～20分=极严重功能障碍，20～45分=严重功能障碍，50～70分=中度功能障碍，75～95分=轻度功能障碍，100分=ADL自理。
2. 术前衰弱评估：改良衰弱指数 mFI( modified frailty index) 是CSHA-FI的简化形式，将mFI评分0分为健康，0～0.21分为衰弱前状态，≥0.21分为衰弱状态。包括11项：非独立功能或活动状态；糖尿病史；COPD病史或肺炎；充血性心力衰竭病史；心肌梗死病史；心绞痛/PCI术后/心脏手术后；高血压需要药物治疗；外周血管疾病或静态痛；感觉障碍；TIA发作或无后遗症的脑血管意外；脑血管意外伴后遗症。

在本研究中,BI≤60分中重度功能障碍病人被认为是项目1为阳性。所有为阳性的项目数累积，除以被评估的项目总数以获得mFI量表评分。

1. 患者的基线资料、既往史等。
2. 术前认知评估：简易精神状态评估量表（MMSE）
3. 年龄校正查尔森合并症指数（aCCI）评估术前合并症。

**十一、术中数据**

1. 手术细节，包括手术的类型和时间；
2. 麻醉细节，包括使用的任何药物的名称和剂量；
3. 血管活性药和正性肌力药的名称和剂量；
4. 所用液体的名称和容量，估计的失血量，尿量和血液制品。

**十二、术后数据**

精神科医生将对随访人员进行培训3D-CAM的使用方法。

1. 术后谵妄：使用3D-CAM在术后第1至3天每天评估两次（上午8:00-10:00，下午18:00-20:00）。
2. 住院时间：将主治医师认为可以出院的时间作为住院的最后一天。
3. 其他并发症：术后30天内发生的任何并发症（心脏事件、脑血管事件、肾损伤、感染等）。

**十三、主要研究结局**

主要结局是非心脏手术术后谵妄的发生率

**十四、次要研究结局**

1. 30天再次入院的发生率；
2. 术后随访患者30天内并发症的发生情况，包括肺部感染、尿路感染、心脑血管意外、肝功能异常、术后出血、切口感染、下肢深静脉血栓形成、电解质紊乱、低蛋白血症；
3. ICU时间和住院时间。

**十五、不良事件**

1）不良事件定义为任何与医疗干预相关且不可预测的、不利的医疗事件。它可能与研究方案或其他方面有关，它可以表现为任何不舒服的症状，也包括异常的实验室检查结果，短期的并发症；

2）本研究中的不良事件和处理：

- 心动过速：心率 > 100次/分，或者如果基线值 > 83次/分则比基线增加20％以上；给予艾司洛尔或地尔硫卓和/或调整麻醉剂量；
- 高血压：收缩压 > 160 mmHg，或者如果基线值 > 133 mmHg则比基线增加20％以上；给予乌拉地尔或硝酸甘油和/或调整麻醉剂量；
- 心动过缓：心率 < 55次/分，或者如果基线值 < 69次/分则比基线减少20％以上；静脉给予阿托品和/或调整麻醉剂量；
- 低血压：收缩压 < 95 mmHg，或者如果基线值 < 119 mmHg则从基线下降超过20％；静脉输注液体，给予血管加压药物，和/或调整麻醉剂量；
- 术中知晓：在适当的全身麻醉和标准治疗期间，患者可以回忆起术中的事件。

3）应记录任何不良事件，包括类型、时间、持续时间、处理方式和后遗症；任何不良事件都应该持续随访，直到完全解决或治疗终止；

4）严重不良事件为任何导致死亡、生命危险、住院时间延长、持续残疾或功能障碍或其他不可预测的严重医疗事件。如果出现任何严重不良事件，将停止研究方案并立即开始治疗；

5）如果主治麻醉医师或研究人员认为必要，可暂时或永久停止研究方案。 研究中断的时间和原因将记录在病例记录表（CRF）中；

6）如有任何严重不良事件，除上述积极治疗和记录外，在24小时内以书面报告通知主要研究者和伦理委员会；

7）如果发现研究药物相关的死亡，立即停止临床试验向伦理委员会报告，详细记录并保存相关文件。

**十七、数据管理**

1）研究人员应根据原始观察结果及时、完整、正确地在CRF表中记录数据；

2）研究协调员将监管研究是否按照方案进行， 经项目总负责人签署后，已完成的CRF将发送给负责数据管理的研究人员；

3）由一名研究人员执行数据输入，另一研究人员检查。 CRF将按顺序进行存储；

4）伦理委员会可以随时检查数据管理情况。

**十八、统计分析**

1. 对连续变量（如年龄等）进行正态性检验，若服从正态分布则以平均数±标准差表示，组间比较采用独立样本t检验；若不服从正态分布，则以中位数（四分位数间距）表示，组间比较采用wilcoxon秩和检验；分类变量（如性别，并发症等）将以频数（百分比）表示，组间比较采用卡方检验或Fisher精确概率法。
2. 采用Logistic回归模型对主要结果术后谵妄进行分析，计算优势比（OR）和95％可信区间（CI）评价衰弱与术后谵妄的关系。
3. 采用Kaplan-Meier生存曲线法描述次要结局的发生率，以log-rank检验比较组间差异，采用Cox回归模型分析30天再次入院、30天内并发症发生情况的影响因素。
4. 采用卡方检验比较两组之间病例脱落率。
5. 双侧检验将用于所有统计分析，P值小于0.05为差异具有统计学意义。

**十九、质量控制（研究者）**

1）在试验开始前，将向所有研究者/护理人员详细解释试验方案。 在整个试验期间必须严格遵守试验方案；

2）准确地记录所有预期和意外的结果，以保证数值的可靠性；

3）研究期间使用的监护器和其他仪器将定期检查和校正，以保证其正常运行；

4）统计人员和研究人员将进行严格的数据分析；

5）所有结论必须由原始数据中得出。

**二十、质量控制（受试者）**

1）将向每个潜在的受试者清楚地解释与研究相关的可能的益处和风险；

2）每位入组的受试者或授权代理人必须签署书面知情同意书；

3）如果入组的受试者在研究期间拒绝参与该研究，则将排除该受试者进一步的研究；

4）如果在研究期间发生与研究相关的死亡，研究将停止。相关报告将发送给道德委员会。重新开始研究需要得到伦理委员会的批准。

5）在完成受试者招募和数据收集后，研究将终止。将由研究人员做出决定。

**二十一、伦理和知情同意**

1）研究方案必须经过伦理委员会的批准才能开始研究；

2）必须向每位受试者提供书面知情同意书。每位参与者或授权代理人必须在他们参加研究之前签署同意书，书面知情同意书将作为临床试验文件的一部分保管。
