# Supplementary material for: Correlation of preoperative frailty with postoperative delirium and one-year mortality in Chinese geriatric patients undergoing noncardiac surgery: Study protocol for a prospective observational cohort study
Source: PLoS One. 2024 Mar 6;19(3):e0295500. doi: 10.1371/journal.pone.0295500 (PMC10917300; doi:10.1371/journal.pone.0295500)
Supplement: S2 File — (DOC) [file pone.0295500.s002.doc]

**研究计划书**

**中国老年非心脏手术患者术前虚弱与术后谵妄和1年死亡率的相关性：一项单中心、前瞻性、观察性、队列研究**

**Correlation of preoperative frailty with postoperative delirium and one-year mortality in Chinese geriatric undergoing noncardiac surgery patients: study protocol for a prospective observational cohort study**

**负责单位： 山东第一医科大学第一附属医院**

**研究负责人：山东第一医科大学第一附属医院麻醉与围术期医学科**

**副主任医师 孙永涛**

**版本号：第四版 2021-11-8**

**Correlation of preoperative frailty with postoperative delirium and one-year mortality in Chinese geriatric undergoing noncardiac surgery patients: study protocol for a prospective observational cohort study**

1. **Background**

As a country with the largest population in the world, China's aging population is also one of the fastest developing countries. It is estimated that by 2025, there will be nearly 300 million people over the age of 65. By 2050, the country will have 400 million elderly people. The aging problem is getting worse. More importantly, the number of elderly people undergoing surgery has increased faster than the aging of the population in the past 20 years [1]. In addition, studies have shown that frailty is more common in the surgical population (42%-50% frailty) than in the non-surgical elderly population (4%-10% frailty). Therefore, preoperative evaluation of the comprehensive health status of elderly patients and early reversal or alleviation of frailty state are extremely urgent [2]. Because every year, more than 500,000 patients over the age of 65 undergo low-risk and high-risk surgery [3].

Frailty is a non-specific condition in the elderly with decreased ability to resist stress due to decreased physiological reserve. Frailty involves physiological changes of multiple systems, including neuromuscular system, metabolic system and immune system. Is a complex, multifaceted, periodic state of reduced physiological reserves, resulting in decreased resilience and adaptability, and increased vulnerability to stressors. This state increases the risk of negative events such as death, disability, delirium and falls [4]. Frailty is associated with adverse perioperative outcomes and is considered a risk factor for the development of certain cardiovascular diseases, such as hypertension, heart failure, and ischemic heart disease. Frailty is common in elderly patients undergoing surgery [5,6,7]. Among the elderly over 70 years old, 38%-54% people have frailty, and the comprehensive frailty is 35%-41%.

Postoperative delirium (POD) is a common complication, which usually occurs between 24 hours and 72 hours after surgery and affects 20% to 80% of elderly surgical patients [8]. At present, it is known that postoperative delirium POD has a variety of risk factors that can be intervened or not intervened, such as age, type of surgery, admission to ICU, pain and certain drugs. Age is one of the major risk factors that cannot be intervened. Therefore, the guidelines recommend that elderly surgical patients receive preoperative screening to screen for geriatric diseases related to postoperative delirium and poor surgical results [9]. Among them, the most important is weakness, which is also a powerful predictor of a variety of adverse health outcomes, including falls, disability and dementia in the elderly [10,11]. At the same time, weakness is also an independent predictor of surgical prognosis [12], and has been proved to be the most common disease leading to death of the elderly [13], which shows that it is important to identify the weakness of elderly patients in clinical practice. Therefore, early identification of debilitating patients has important clinical significance for preoperative decision-making and prognosis evaluation. So far, the incidence of postoperative delirium in elderly patients undergoing noncardiac surgery has not been evaluated. This study intends to explore the relationship between preoperative asthenia assessment and the incidence of postoperative delirium after noncardiac surgery.

**References**

1. Kulason Kay,Nouchi Rui,Hoshikawa Yasushi et al. Indication of Cognitive Change and Associated Risk Factor after Thoracic Surgery in the Elderly: A Pilot Study.[J] .Front Aging Neurosci, 2017, 9: 396.
2. Fried L P,Tangen C M,Walston J et al. Frailty in older adults: evidence for a phenotype.[J] .J Gerontol A Biol Sci Med Sci, 2001, 56: M146-56.
3. Schwarze ML, Barnato AE, Rathouz PJ, Zhao Q, Neuman HB, Winslow ER, Kennedy GD, Hu YY, Dodgion CM, Kwok AC, Greenberg CC. Development of a list of high-risk operations for patients 65 years and older. JAMA Surg. 2015 Apr;150(4):325-31. doi: 10.1001/jamasurg.2014.1819. PMID: 25692282; PMCID: PMC4414395.
4. Clegg A，Young J，Iliffe S，et al. Frailty in elderly people［J］. Lancet，2013，381（9868）： 752-762. DOI： 10.1016/S0140-6736（12）62167-9.
5. Woo J, Yu R, Wong M, Yeung F, Wong M, Lum C: Frailty screening in the community using the FRAIL scale. J Am Med Dir Assoc. 2015; 16:412–9
6. Gleason LJ, Benton EA, Alvarez-Nebreda ML, Weaver MJ, Harris MB, Javedan H: FRAIL questionnaire screening tool and short-term outcomes in geriatric fracture patients. J Am Med Dir Assoc. 2017; 18:1082–6
7. Aprahamian I, Cezar NOC, Izbicki R, Lin SM, Paulo DLV, Fattori A, Biella MM, Jacob Filho W, Yassuda MS: Screening for frailty with the FRAIL scale: A comparison with the phenotype criteria. J Am Med Dir Assoc. 2017; 18:592–6.
8. Inouye SK. Delirium in older persons. N Engl J Med. 2006 Mar 16;354(11):1157-65. doi: 10.1056/NEJMra052321.
9. Chow WB, Rosenthal RA, Merkow RP, Ko CY, Esnaola NF; American College of Surgeons National Surgical Quality Improvement Program; American Geriatrics Society. Optimal preoperative assessment of the geriatric surgical patient: a best practices guideline from the American College of Surgeons National Surgical Quality Improvement Program and the American Geriatrics Society. J Am Coll Surg. 2012 Oct;215(4):453-66. doi: 10.1016/j.jamcollsurg.2012.06.017. Epub 2012 Aug 21. PMID: 22917646.
10. Aldecoa C, Bettelli G, Bilotta F, Sanders RD, Audisio R, Borozdina A, Cherubini A, Jones C, Kehlet H, MacLullich A, Radtke F, Riese F, Slooter AJ, Veyckemans F, Kramer S, Neuner B, Weiss B, Spies CD. European Society of Anaesthesiology evidence-based and consensus-based guideline on postoperative delirium. Eur J Anaesthesiol. 2017 Apr;34(4):192-214. doi: 10.1097/EJA.0000000000000594. Erratum in: Eur J Anaesthesiol. 2018 Sep;35(9):718-719. PMID: 28187050.
11. Dent E，Martin FC，Bergman H，et al． Management of frailty: opportunities，challenges，and future directions［J］． Lancet，2019，394 ( 10206) : 1376-1386．
12. Amini S, Crowley S, Hizel L, Arias F, Libon DJ, Tighe P, Giordano C, Garvan CW, Enneking FK, Price CC. Feasibility and Rationale for Incorporating Frailty and Cognitive Screening Protocols in a Preoperative Anesthesia Clinic. Anesth Analg. 2019 Sep;129(3):830-838. doi: 10.1213/ANE.0000000000004190. PMID: 31425227; PMCID: PMC6927245.
13. Gill TM, Gahbauer EA, Han L, Allore HG. Trajectories of disability in the last year of life. N Engl J Med. 2010 Apr 1;362(13):1173-80. doi: 10.1056/NEJMoa0909087. PMID: 20357280; PMCID: PMC2877372.
14. **Study objective**

1) To investigate the correlation between frailty and postoperative delirium in elderly patients undergoing non-cardiac surgery

2) Can modified frailty index predict delirium after non-cardiac surgery

3) What are the independent risk factors of postoperative delirium in frailty index

**三、Inclusion criteria**

1) Age ≥70 years;

2)ASA: Ⅰ～Ⅳ;

3) Sign the informed consent;

4) Plan elective non-cardiac surgery.

**四、Exclusion criteria**

1) Refuse to participate;

2) Expected length of hospital stay & LT; 3 days;

3) The same patient can only be included once, regardless of whether the reason for the second operation is related to the first cause;

4) Emergency operation patients;

5) Unable to communicate because of illiteracy, language impairment, severe hearing or visual impairment;

6) Central nervous system diseases, including various types of dementia, depression;

7) Severe renal insufficiency (requiring dialysis treatment);

8) Severe abnormal liver function (Child-Pugh score ≥10);

9) Patients who have participated in other relevant clinical studies within 3 months

10)MMSE examination confirmed the existence of cognitive dysfunction: illiteracy ≤17 points, primary school ≤20 points, secondary school (including technical secondary school) ≤22 points, university (including junior college) ≤23 points.

**五、Abscission criterion**

The patient or client refused informed consent or requested withdrawal from the study.

1. **Study type**

The study was a single-center, prospective, observational, cohort study

1) For each recruited patient, each investigator completed preoperative assessment 1 day before surgery: frailty screening, cognitive function assessment, and preoperative data collection;

2) According to the division of preoperative visit, each researcher will be responsible for intraoperative index collection and specimen collection (pre-operation and post-operation);

3) Another 3 researchers will be responsible for 1-3D postoperative follow-up and data collection;

4) Statistical analyses will be performed independently by another designated statistician.

**七、**Sample size

According to the pre-experiment, the incidence of frailty in non-cardiac surgery patients aged ≥70 years was 23%. The incidence of POD was 35.7% in frailty group and 21.7% in non-frailty group. The sample size calculation method of the independent sample rate comparison between the two groups using PASS 15 software: The sample size ratio between the experimental group and the control group was 0.303. The test level (α) and test power (1-β) were set as 0.05 and 0.80, respectively. Considering the 10% shedding rate, the sample size of the frailty group was 114 cases and that of the control group was 375 cases, a total of 489 patients.

八、Anesthesia management

Anesthesia induction was performed with liyuexin 0.05-0.1 mg/kg, propofol 1.5-2 mg/kg or etomidate 0.15-0.3mg/kg, sufentanil 0.3-0.5μg/kg, rocuronium 0.6 mg/kg or cis-atracurium 0.15 mg/kg. Anesthesia was maintained with propofol 1.5-4.0μg/mL, opioids were maintained with remifentanil 0.1-2μg/(kg·min), and muscle relaxants were maintained with rocuronium 5-10μg/kg/min. The end-respiratory partial pressure of CO2 was maintained at 35-45 mmHg, and the depth of anesthesia with Bispectral index (BIS) was maintained at 40-60. Ondansetron 8mg was given intraoperatively. Postoperative patient-controlled intravenous analgesia (PCIA, sufentanil 0.08-0.1μg/kg/h, the total amount controlled within 150-250 μg) was administered.

1. **Preoperative data**

Each researcher carefully completed the frailty assessment and the assessment of the ability of daily living (see Annex 1 for the scoring criteria).

1) Assessment of the ability of daily living: the Barthel index was used to assess the ability of daily living of patients one day before surgery; Score 0-20 = very severe dysfunction, score 20-45 = severe dysfunction, score 50-70 = moderate dysfunction, score 75-95 = mild dysfunction, score 100 = self-care in ADL.

2) Preoperative frailty assessment: Modified frailty Index (mFI) is a simplified form of CSHA-FI. MFI score 0 is classified as healthy, 0-0.21 as pre-frailty, and ≥0.21 as frailty. Including 11 items: non-independent function or active state; History of diabetes; A history of COPD or pneumonia; A history of congestive heart failure; History of myocardial infarction; Angina pectoris/after PCI/after cardiac surgery; High blood pressure requires medication; Peripheral vascular disease or static pain; A sensory disorder; TIA or cerebrovascular accident without sequelae; Cerebrovascular accident with sequelae.

In this study, patients with moderate to severe dysfunction with BI≤60 were considered positive for item 1. The number of all positive items was accumulated and divided by the total number of items evaluated to obtain the mFI scale score.

3) Baseline data and past history of patients.

4) Preoperative cognitive assessment: Mini-Mental State Assessment (MMSE)

5) Age-adjusted Charlson comorbidity Index (aCCI) was used to evaluate preoperative comorbidity.

**十一、Intraoperative data**

1. Surgical details, including type and time of surgery;
2. Anesthetic details, including the name and dosage of any drug used;
3. Names and dosages of vasoactive drugs and inotropic drugs;
4. Name and volume of fluid used, estimated blood loss, urine volume, and blood products.

**十二、Postoperative data**

1. Psychiatrists will train follow-up staff on the use of 3D-CAM.
2. Postoperative delirium: 3D-CAM was used to evaluate twice daily (8:00-10:00 am, 18:00-20:00 PM) on postoperative days 1 to 3.
3. Length of hospital stay: the last day of hospital stay will be the time that the attending physician thinks can be discharged.
4. Other complications: any complications (cardiac events, cerebrovascular events, renal injury, infection, etc.) occurring within 30 days after surgery.

**十三、Primary outcome**

Incidence of delirium after noncardiac surgery

**十四、Secondary outcomes**

1. The incidence of 30-day readmission;
2. The incidence of complications within 30 days of postoperative follow-up, including pulmonary infection, urinary tract infection, cardiovascular and cerebrovascular accident, abnormal liver function, postoperative bleeding, incision infection, deep vein thrombosis of lower extremities, electrolyte disturbance, and hypoproteinemia;
3. ICU duration and hospital stay.

**十五、Adverse events**

1) An adverse event is defined as any unpredictable and adverse medical event related to a medical intervention. It can be protocol-related or otherwise. It can manifest as any number of uncomfortable symptoms. It can also include abnormal laboratory results, short-term complications;

2) Adverse events and management in this study:

Tachycardia: Heart rate & GT; 100 beats/min, or if the baseline value & GT; 83 times per minute is more than 20% more than the baseline; Administration of esmolol or diltiazem and/or adjustment of anesthetic dose;

Hypertension: Systolic blood pressure & GT; 160 mmHg, or if baseline & GT; 133 mmHg was an increase of more than 20% from baseline; Administration of urapidil or nitroglycerin and/or adjustment of anesthetic dose;

Bradycardia: Heart rate & LT; 55 beats/min, or if baseline & LT; 69 times per minute is a reduction of more than 20% from baseline; Intravenous administration of atropine and/or adjustment of anesthetic dose;

Hypotension: Systolic blood pressure & LT; 95 mmHg, or if baseline & LT; 119 mmHg decreased by more than 20% from baseline; Intravenous fluids, administration of vasopressors, and/or adjustment of the anesthetic dose;

Intraoperative awareness: The patient can recall the events of the procedure during appropriate general anesthesia and standard care.

3) Any adverse events should be recorded, including type, time, duration, treatment and sequelae; Any adverse events should be followed up until complete resolution or treatment termination;

4) Serious adverse events are any serious medical events that result in death, life risk, prolonged hospital stay, persistent disability or dysfunction, or other unpredictable events. If any serious adverse events occur, the study protocol will be discontinued and treatment will begin immediately;

5) The study protocol may be temporarily or permanently discontinued if the attending anesthesiologist or investigator deems it necessary. The duration and cause of the study interruption will be recorded in the Case Notes Form (CRF);

6) Notify the principal investigator and the ethics committee of any serious adverse events in a written report within 24 hours in addition to the above active treatment and documentation;

7) If death related to the study drug is found, the clinical trial shall be immediately stopped and reported to the ethics committee, with detailed records and relevant documents kept.

**十七、Data management**

1) Researchers should record data in CRF tables in a timely, complete and correct manner according to the original observations;

2) The research coordinator will supervise whether the study is carried out in accordance with the protocol, and the completed CRF will be sent to the researcher in charge of data management after it is signed by the general manager of the project;

3) Data entry was performed by one researcher and checked by another researcher. CRF will store in order;

4) The ethics committee can check the data management at any time.

**十八、Statistical analysis**

1. Normality test was performed on continuous variables (such as age, etc.). If they were normally distributed, they were expressed as mean ± standard deviation, and independent sample t-test was used for comparison between groups. If normal distribution was not followed, the median (interquartile range) was expressed. Wilcoxon rank sum test was used for comparison between groups. Categorical variables (e.g., sex, complications, etc.) will be expressed as frequencies (percentages), and comparisons between groups will be performed using the chi-square test or Fisher's exact test.
2. Logistic regression model was used to analyze the primary outcome of postoperative delirium. Odds ratio (OR) and 95% confidence interval (CI) were calculated to evaluate the relationship between frailty and postoperative delirium.
3. Kaplan-Meier survival curve method was used to describe the incidence of secondary outcomes, Log-rank test was used to compare the differences between groups, and Cox regression model was used to analyze the influencing factors of 30-day readmission and complications within 30 days.
4. Chi-square test was used to compare the case dropping rate between the two groups.
5. Two-sided test will be used for all statistical analyses, and P value less than 0.05 is considered statistically significant.

**十九、Quality Control (Researcher)**

1) The protocol will be explained in detail to all investigators/caregivers prior to the start of the trial. The test protocol must be strictly adhered to throughout the test period;

2) Accurately record all expected and unexpected results to ensure the reliability of numerical values;

3) Monitors and other instruments used during the study will be checked and corrected regularly to ensure their normal operation;

4) Statisticians and researchers will conduct rigorous data analysis;

5) All conclusions must be drawn from the original data.

**二十、Quality Control (Subject)**

1) The possible benefits and risks associated with the study will be clearly explained to each potential subject;

2) Each enrolled subject or authorized agent must sign written informed consent;

3) If the enrolled subject refuses to participate in the study during the study period, the subject will be excluded from further study;

4) If a study-related death occurs during the study period, the study will be discontinued. A report will be sent to the ethics committee. Ethics Committee approval is required to restart the study.

5) The study will be terminated upon completion of subject recruitment and data collection. It will be up to the researchers to decide.

**二十一、Ethical and informed consent**

1) The study protocol must be approved by the ethics committee before starting the study;

2) Written informed consent must be provided to each subject. Each participant or authorized agent must sign the consent form prior to their participation in the study, and the written informed consent form will be kept as part of the clinical trial documentation.
