# Supplementary material for: Correlation of preoperative frailty with postoperative delirium and one-year mortality in Chinese geriatric patients undergoing noncardiac surgery: Study protocol for a prospective observational cohort study
Source: PLoS One. 2024 Mar 6;19(3):e0295500. doi: 10.1371/journal.pone.0295500 (PMC10917300; doi:10.1371/journal.pone.0295500)
Supplement: Attachment — Submitted filename: Response to Editor 5.11.docx [file pone.0295500.s003.docx]

Dear Prof. Rose Ann Joyce Sagun Puetes:

Thank you for your letter and for your comments concerning our manuentitled “Correlation of preoperative frailty with postoperative delirium and one-year mortality in Chinese geriatric patients undergoing noncardiac surgery: a prospective observational cohort study” (ID: PONE-D-22-32948R1). Those comments are all valuable and very helpful for revising and improving our paper, as well as the important guiding significance to our researches. We have studied comments carefully and have made correction which we hope meet with approval. Revised portion are marked in red in the paper.

The main corrections in the paper and the responds to the reviewer’s comments are as flowing:

**Journal Requirements:**

We've checked your submission and before we can proceed, we need you to address the following issues:

1. Thank you for submitting your manuscript to PLOS ONE and for responding to our recent requests regarding your submission. Please accept our sincere apologies for the delay in responding to your query. As previously indicated, the CONSORT flow diagram in Figure 2 should be removed. This flow chart is used to show the number of participants excluded/included in the final analysis of a clinical trial. Since your manuscript describes work not yet completed, information on the number of participants included in the analysis cannot be provided. For this reason, it is not appropriate to include a CONSORT diagram in your manuscript. You may include a flow diagram to demonstrate your study design, but a CONSORT diagram is not appropriate for the purpose.

We suggest removing the flow diagram and relying on text in the Methods to describe the study design. Alternatively, you may provide a new diagram to illustrate your study design, but this should be based on the CONSORT diagram.

Response 1 We gratefully appreciate for your valuable suggestion/ comment. We have made changes based on your comments. We have already removed the CONSORT flow diagram. We have modified the "CONSORT flowchart" to the "Flow diagram of the study" and redone the diagram. We don't know if this modification meets the requirements. Can you give us some advice?

**Fig. 2** Flow diagram of the study

In addition, one of our coauthors had a change of institution. I have changed the author's information, please know.

Min Zhang^1†^, Xiaojun Gao^1†^, Mengjie Liu^1^, Zhongquan Gao^1,3^, Xiaxuan Sun^1,3^, Linlin Huang^4^, Ting Zou^4^, Yongle Guo^1,3^, Lina Chen^1^, Yang Liu^1^, Xiaoning Zhang^1^, Hai Feng^1^, Yuelan Wang^2,3^, Yongtao Sun^1^*

^1^ Department of Anesthesiology, The First Affiliated Hospital of Shandong First Medical University & Shandong Provincial Qianfoshan Hospital, Shandong Institute of Anesthesia and Respiratory Critical Medicine, Jinan 250014, China

^2^ Department of Anesthesiology, Provincial Hospital Affiliated to Shandong First Medical University (Shandong Provincial Hospital), Jinan 250014, China

^3^ Department of Anesthesiology, Shandong First Medical University, Jinan 250014, China

^4^ Department of Nursing, The First Affiliated Hospital of Shandong First Medical University & Shandong Provincial Qianfoshan Hospital, Jinan 250014, China

*Corresponding author:

Yongtao Sun, Phone: +8618660795201, Email: ytsun@sdfmu.edu.cn

^†^These authors also contributed equally to this work.
