# Supplementary material for: Correlation of preoperative frailty with postoperative delirium and one-year mortality in Chinese geriatric patients undergoing noncardiac surgery: Study protocol for a prospective observational cohort study
Source: PLoS One. 2024 Mar 6;19(3):e0295500. doi: 10.1371/journal.pone.0295500 (PMC10917300; doi:10.1371/journal.pone.0295500)
Supplement: Attachment — Submitted filename: Response to Editor.docx [file pone.0295500.s004.docx]

Dear Prof. Academic Editor:

Thank you for your letter and for your comments concerning our manuentitled “Correlation of preoperative frailty with postoperative delirium and one-year mortality in Chinese geriatric patients undergoing noncardiac surgery: a prospective observational cohort study” (ID: PONE-D-22-32948R1). Those comments are all valuable and very helpful for revising and improving our paper, as well as the important guiding significance to our researches.

**ACADEMIC EDITOR:**

I have specific concerns that this is a study protocol of a cohort study, but the authors have included elements of a clinical trial. The authors have included a study flowchart (Figures 2) that is still not suitable as they include numbers of participants or (n= ). This is not appropriate. If you include a flowchart it should only explain your study design. Please revise it accordingly. Alternatively, please remove Figure 2.

**Response 1:** Thank you for the constructive comments and suggestions. We have removed Figure 2.

In addition, in the revised version you do not appear to have provided a response specifically to the original reviewers’ comments. Please ensure that you provide this and a suitably marked up version of the manuscript highlighting how you have responded to all of the points raised (particularly by reviewer #1 in the original review).

**Response 2:** We feel sorry for the inconvenience brought to you. We previously responded individually to the comments of the three reviewers. It was later removed. According to your requirements, we have uploaded the file in “Response to Reviewers” again, the file name is Response, please review.
