# Supplementary material for: Correlation of preoperative frailty with postoperative delirium and one-year mortality in Chinese geriatric patients undergoing noncardiac surgery: Study protocol for a prospective observational cohort study
Source: PLoS One. 2024 Mar 6;19(3):e0295500. doi: 10.1371/journal.pone.0295500 (PMC10917300; doi:10.1371/journal.pone.0295500)
Supplement: Attachment — Submitted filename: Response to Editor9.07.docx [file pone.0295500.s005.docx]

Dear Academic Editor Silvia Fiorelli:

Thank you for your letter and for your comments concerning our manuentitled “Correlation of preoperative frailty with postoperative delirium and one-year mortality in Chinese geriatric patients undergoing noncardiac surgery: a prospective observational cohort study” (ID: PONE-D-22-32948R1). Those comments are all valuable and very helpful for revising and improving our paper, as well as the important guiding significance to our researches.

**ACADEMIC EDITOR:**

Thank you for carefully assess all reviewers points.

I still have a specific comment on figure 2.

Since your manuscript describes work not yet completed, information on the number of
participants included in the analysis cannot be provided.

so you should remove all the patients numbers in this figure and leave " (n=)".

**Response 1:** Thank you for the constructive comments and suggestions. We have removed remove all the patients numbers in this figure and leave " (n=)".
